# Supplementary material for: Using interpretable machine learning to predict bloodstream infection and antimicrobial resistance in patients admitted to ICU: Early alert predictors based on EHR data to guide antimicrobial stewardship
Source: PLOS Digit Health. 2024 Oct 16;3(10):e0000641. doi: 10.1371/journal.pdig.0000641 (PMC11482717; doi:10.1371/journal.pdig.0000641)
Supplement: S1 Appendix — When the equations are of reasonable complexity, we report it below, otherwise we omit them in the manuscript. Variables need to be scaled between 0 and 1. (DOCX) [file pdig.0000641.s001.docx]

**S1 Appendix – Models and confusion matrices**

When the equations are of reasonable complexity, we report it below, otherwise we omit them in the manuscript. Variables need to be scaled between 0 and 1.

***BSI prediction on real-world data***

***Model with best AUC***

$$Bilirubin+\left( MeanBloodPressure*log\left( Urea \right) \right)+{PaO}_{2}*\left( {Bicarbonate}^{-1.04}-4.22*ABUsed \right)$$

| Features used - 6/24 | Predicted Negative | Predicted Positive |
| --- | --- | --- |
| True Negative | 89 | 13 |
| True Positive | 6 | 7 |

***Model with best F1 Score***

$$0.58*{Bicarbonate}^{-0.88}-2.38*Sodium-1.63*ABUsed$$

| Features used - 3/24 | Predicted Negative | Predicted Positive |
| --- | --- | --- |
| True Negative | 78 | 24 |
| True Positive | 4 | 9 |

***BSI prediction on augmented data***

***Model with best AUC***

$$\sqrt{log\left( Temperature \right)+log\left( \sqrt{log\left( ABUsed \right)} \right)}$$

| Features used - 2/24 | Predicted Negative | Predicted Positive |
| --- | --- | --- |
| True Negative | 18 | 84 |
| True Positive | 1 | 12 |

***Model with best F1 Score***

*(complex equation)*

| Features used - 22/24 | Predicted Negative | Predicted Positive |
| --- | --- | --- |
| True Negative | 84 | 18 |
| True Positive | 6 | 7 |

***AMR prediction on real-world data***

***Model with best AUC and F1 Score***

$${Nystatin}_{100.000U/ml}+log\left( MeanBloodPressureScore+{Bilirubin}^{ABUsed} \right)$$

| Features used – 4/24 | Predicted Negative | Predicted Positive |
| --- | --- | --- |
| True Negative | 80 | 24 |
| True Positive | 1 | 10 |

***AMR prediction on augmented data***

***Model with best AUC***

*(complex equation)*

| Features used – 11/24 | Predicted Negative | Predicted Positive |
| --- | --- | --- |
| True Negative | 29 | 75 |
| True Positive | 0 | 11 |

***Model with best F1 Score***

*(complex equation)*

| Features used – 16/24 | Predicted Negative | Predicted Positive |
| --- | --- | --- |
| True Negative | 90 | 14 |
| True Positive | 6 | 5 |
