# Supplementary material for: Using interpretable machine learning to predict bloodstream infection and antimicrobial resistance in patients admitted to ICU: Early alert predictors based on EHR data to guide antimicrobial stewardship
Source: PLOS Digit Health. 2024 Oct 16;3(10):e0000641. doi: 10.1371/journal.pdig.0000641 (PMC11482717; doi:10.1371/journal.pdig.0000641)
Supplement: Attachment — Submitted filename: Article REVIEW.pdf [file pdig.0000641.s002.pdf]

Article Title: "Using interpretable Machine Learning to predict bloodstream infection and antimicrobial resistance in patients admitted to ICU: early alert predictors based on EHR data to guide antimicrobial stewardship".

Abstract

The abstract provides a clear overview of the study's purpose, methods, and significant findings. It successfully outlines the novelty and relevance of using Multi-Objective Symbolic Regression (MOSR) for predicting bloodstream infections (BSI) and Antimicrobial Resistance (AMR) in ICU patients. The comparison of MOSR with traditional ML models and the emphasis on its ability to minimize false negatives are well-presented.

Introduction

The introduction effectively sets the context of the problem, discussing the impact of nosocomial infections and AMR on healthcare. The justification for using ML techniques, particularly MOSR, is well-articulated. However, the introduction could be improved by briefly mentioning previous studies in this domain to establish a clearer research gap.

Methodology

The methodology section is comprehensive, detailing the dataset, ML models used, and the rationale behind choosing MOSR. The explanation of the feature selection process and the definition of BSI and AMR as binary outcomes are well-explained. However, a more detailed discussion on the selection criteria for the initial dataset of over 5000 patients could enhance transparency. Additionally, elaborating on the specific types of data included (e.g., types of clinical data used) would provide more insight into the study's applicability.

Results

The results section effectively presents epidemiological analyses and comparative performance metrics of various ML models. The finding that MOSR outperforms other models in both balanced and imbalanced scenarios is significant. However, this section could benefit from a more detailed discussion on why certain models performed better than others, possibly including more technical details or insights into the features that most influenced model performance.

Discussion and Conclusion

The discussion adequately highlights the implications of the study, emphasizing the potential of MOSR in a clinical setting. The acknowledgment of limitations such as data scarcity and imbalance is commendable, as it indicates areas for future research. To strengthen this section, the authors could consider comparing their findings with similar studies and discussing potential real-world applications and challenges in implementing their model in clinical settings.

Recommendations for Improvement:

1. Literature Contextualization: Enhance the introduction by including a brief review of similar studies to establish a more defined research gap.
2. Dataset Details: Provide more specifics about the initial dataset selection and the types of clinical data used.
3. Model Performance Analysis: In the results section, include a deeper analysis of why MOSR performed better, focusing on the features and model characteristics that contributed to its success.
4. Comparative Discussion: Compare the findings with other similar studies in the discussion to contextualize the study's contribution to the field.
5. Implementation Insights: Discuss the practical challenges and potential strategies for implementing the proposed ML model in real-world clinical settings.

Analysis

1. Research Context and Novelty:
   - The manuscript successfully contextualizes the challenge of nosocomial infections and antimicrobial resistance (AMR) in healthcare.
   - It introduces a novel approach using Multi-Objective Symbolic Regression (MOSR) to predict bloodstream infections (BSIs) and AMR, leveraging clinical data collected upon ICU admission.
2. Methodology:
   - The study's methodology, involving data from over 5000 ICU patients, is robust, with a clear explanation of the data selection process and feature set reduction using HSIC Lasso .
   - The choice of ML models, including Logistic Regression, Decision Trees, Random Forests, Gradient Boosting, and others, is comprehensive. The justification for not using Deep Learning strategies due to their lack of interpretability is rational.
3. Results:
   - The results demonstrate that MOSR outperforms traditional ML models in predicting BSI and AMR. This is particularly noteworthy in scenarios with imbalanced datasets.
   - The epidemiological analysis revealing the relationship between blood culture requests, BSI incidence, and antibiotic use is insightful and adds value to the study.
4. Discussion and Implications:
   - The discussion effectively highlights the potential of the MOSR approach in clinical settings. It underscores the need for rapid diagnostic support in managing ICU admissions .
   - The acknowledgment of study limitations, including data scarcity and imbalance, reflects a critical and transparent approach.
5. General Observations:
   - The writing is clear and well-structured, making complex concepts accessible.

- The study is grounded in a significant real-world problem, enhancing its relevance and impact.

Recommendations for Improvement

1. Expand Literature Review:
    - Include a more extensive review of previous studies using ML for BSI and AMR prediction. This could provide a broader context and justify the study's novel contribution more effectively.
2. Detailed Methodological Justification:
    - While the methodology is robust, a more detailed justification for the choice of specific ML models and their configurations would add depth to the understanding of their selection.
3. Enhanced Discussion on Model Interpretability:
    - Given the emphasis on interpretability, a deeper discussion on how MOSR facilitates clinical decision-making compared to less interpretable models would be beneficial.
4. Addressing Data Limitations:
    - Future research directions could include strategies to overcome the data limitations noted, such as collaborating with other institutions for a more extensive dataset.
5. Practical Implementation:
    - Discuss the practical aspects of implementing the MOSR model in clinical settings, including integration with existing healthcare IT systems and clinician training.

Conclusion

The manuscript presents a significant contribution to the field of healthcare analytics, particularly in the critical area of infection control and antimicrobial stewardship in ICU settings. The novel application of MOSR for predicting BSI and AMR using EHR data is a promising advancement. However, enhancing the study with a broader literature context, detailed methodological justifications, and discussions on practical implementation would further strengthen its impact.

Further recommendations:

1. Incorporate Latest Research References: Update the text with the latest references and studies in the field of machine learning applications in healthcare. This will ensure that the content remains current and relevant.
2. Detailed Explanation of MOSR: Expand on the explanation of Multi-Objective Symbolic Regression (MOSR), possibly including a brief overview of its mechanics and advantages over traditional models. This would help readers understand why MOSR is more effective.
3. Graphical Abstract: Create a graphical abstract to visually summarize the study. This can be particularly effective for readers who prefer visual learning and for use in presentations or publications.

4. Patient Impact Focus: Include a section or paragraph that specifically discusses the impact of this research on patient outcomes. This can help to humanize the data and emphasize the real-world implications of the findings.

| Original Text | Correction | Explanation |
|---|---|---|
| Antimicrobial Resistance (AMR) stand | Antimicrobial Resistance (AMR) stands | "Antimicrobial Resistance" is singular, so the verb should be "stands". |
| To address these issues, various infection control protocols and personalized treatment strategies, guided by laboratory tests, aim to detect | To address these issues, various infection control protocols and personalized treatment strategies, guided by laboratory tests, aim at detecting | "Aim at" is the correct phrasal verb for indicating purpose. |
| this study, we introduce a machine learning (ML) approach | this study introduces a machine learning (ML) approach | Maintain consistency in narrative voice; third-person is preferable in academic writing. |
| with the goal of predicting | with the aim of predicting | "Aim" is more formal and suitable for academic writing than "goal". |
| Our findings reveal that traditional ML models exhibit | The findings reveal that traditional ML models exhibit | Use a consistent narrative voice; third-person is preferable in academic writing. |
| subpar performance | suboptimal performance | "Suboptimal" is more formal and appropriate in academic writing than "subpar". |
| MOSR, specifically configured to minimize false negatives, outperforms | MOSR, configured specifically to minimize false negatives, outperforms | Adverb placement: "specifically" should directly modify "configured". |
| Notably, this approach can be readily implemented | Notably, this approach can be implemented readily | Adverb placement: "readily" should be placed after the verb "implemented". |

| | | |
|---|---|---|
| individuals admitted to the Intensive Care Unit (ICU) | individuals admitted to the Intensive Care Unit (ICU) | No correction needed, but maintain consistency in the use of abbreviations. |
| a noteworthy incidence rate of 35% | an incidence rate of 35%, which is noteworthy | Clarity and emphasis on the significance of the incidence rate. |
| and pathogens categorized as DTR (difficult-to-treat and drug-resistant)5–7 | and pathogens categorized as DTR (difficult-to-treat and drug-resistant)[5-7] | Use square brackets for in-text citation numbers for consistency. |
| From the moment a patient is admitted to the ICU | From the moment of a patient's admission to the ICU | Improved phrasing for conciseness and clarity. |
| clinicians often confront challenging decisions | clinicians are often confronted with challenging decisions | Passive voice is more appropriate here to emphasize the situation rather than the agent. |
| These choices become particularly complex when | These decisions become particularly complex when | Consistency in terminology; use "decisions" to align with previous sentence. |
| while awaiting the results of laboratory tests8,9 | while awaiting laboratory test results[8,9] | Improved phrasing and citation format. |
