# Supplementary material for: Using interpretable machine learning to predict bloodstream infection and antimicrobial resistance in patients admitted to ICU: Early alert predictors based on EHR data to guide antimicrobial stewardship
Source: PLOS Digit Health. 2024 Oct 16;3(10):e0000641. doi: 10.1371/journal.pdig.0000641 (PMC11482717; doi:10.1371/journal.pdig.0000641)
Supplement: Attachment — Submitted filename: plos one review.pdf [file pdig.0000641.s004.pdf]

Suggestions for the abstract

1. Consider incorporating a statement on the innovative nature of the MOSR approach to highlight its importance more effectively.
2. Please provide a specific metric or statistic that quantifies the performance improvement of MOSR compared with traditional machine learning models.
3. Consider adding a sentence or two to the potential limitations or future directions for research to provide a more comprehensive overview.
4. Use a more active voice throughout the abstract to make it more engaging and dynamic.

Could you clarify the advantages of the MOSR approach over conventional machine learning techniques in diagnosing bloodstream infections and Antimicrobial Resistance? Furthermore, what might be the ramifications of this method in clinical practice and patient outcomes?

Suggestions for the Introduction

1. Consider adding 1-2 sentences summarizing the study objectives and anticipated contributions/findings.
2. Specify whether "AMR" refers only to antibiotic resistance or includes other multidrug resistance as well.
3. How can we tackle the pressing issue of Antimicrobial Resistance in Intensive Care Units, where the incidence of bloodstream infections reaches a staggering 35%?"
4. Specify whether "AMR" refers only to antibiotic resistance or includes other multidrug resistance as well.

Suggestions for the Methodology

1. Could you elaborate on the methods employed to confirm the discriminatory nature of the features identified via the HSIC Lasso, as it is imperative to ascertain their predictive capabilities and exclude the possibility of overfitting? Furthermore, incorporating an internal validation technique while selecting features would enhance the credibility of claims regarding their relevance.

2. Did you explore techniques to address the class imbalance problem beyond simply weighting the loss function? The results can be improved by addressing the imbalance more aggressively during model training.
3. What methods will be employed to validate and interpret MOSR formulas, and how will clinicians/experts in the field utilize the gathered feedback to determine the applicability and generalizability of the findings?

"The MOSR approach is characterized by innovation, yet it could be further enhanced through the incorporation of established machine learning methodological practices, such as validation, class imbalance mitigation, and clinical evaluation."

Suggestions for the Result section

1. Provide more details on the epidemiological analysis, such as the time period and number of patients. Clarify if Figure 2 is from the study data or other sources.
2. For Table 1, report statistical tests used to compare groups (e.g., t-tests, chi-sq) and indicate which features showed significant differences.
3. Undertake further evaluation using additional metrics beyond AUROC/accuracy, such as precision-recall curves and F1 score plots at various thresholds, particularly for imbalanced classes.
4. Please present the top-performing formulas for the MOSR results to highlight their interpretability. In addition, we evaluated the feature importance derived from these formulas.

5. Discuss the limitations of the MOSR approach for this problem, for example, potential for overfitting, handling class imbalance, and generalizability with limited data.
6. Expand discussion of related work, comparing data/models used. The limitations of this study should be addressed in future research.
7. Suggest validation of new prospective data to assess generalizability and clinical utility before deployment.
8. Connect the results to broader goals and implications for clinical decision-making.

General Question

1. What was the main goal of this study described in the paper?
2. What is Symbolic Regression, and how does it differ from other machine learning algorithms?
3. What is the advantage of using Symbolic Regression in this study?
4. How does this study address the class imbalance issue in a dataset?
5. What is the significance of prioritizing sensitivity in this study?
6. How do the authors validate the performance of machine-learning models?
7. What are the limitations of this study owing to the dataset size?
8. How can we enhance these models in future research?
9. Could you elaborate on the possible clinical implications of the predictive models developed in this study?
10. What is the significance of this research in predicting bloodstream infections and antimicrobial resistance, and how does it relate to existing studies on the topic?
