# Supplementary material for: Using interpretable machine learning to predict bloodstream infection and antimicrobial resistance in patients admitted to ICU: Early alert predictors based on EHR data to guide antimicrobial stewardship
Source: PLOS Digit Health. 2024 Oct 16;3(10):e0000641. doi: 10.1371/journal.pdig.0000641 (PMC11482717; doi:10.1371/journal.pdig.0000641)
Supplement: Attachment — Submitted filename: Response to Reviewers.docx [file pdig.0000641.s003.docx]

Dear Akhilanand Chaurasia
Section Editor
PLOS Digital Health,

Thank you for providing us with the valuable feedback and insightful comments from the reviewers regarding our paper: PDIG-D-23-00418 - "Using Interpretable Machine Learning to Predict Bloodstream Infection and Antimicrobial Resistance in Patients Admitted to ICU: Early Alert Predictors Based on EHR Data to Guide Antimicrobial Stewardship." We appreciate the opportunity to address the concerns raised and to clarify aspects of our research.

Reviewer #1: The article presents a study that introduces a machine learning approach based on Multi-Objective Symbolic Regression (MOSR) for predicting bloodstream infections (BSI) and antimicrobial resistance (AMR) in ICU patients using electronic health record (EHR) data. The study compares MOSR with traditional machine learning models, highlighting its superior performance in minimizing false negatives and its potential for large-scale implementation to guide antimicrobial stewardship. There are several issues with this article that need improvement:

- The binary outcomes for BSI and AMR can introduce bias in the machine learning models due to class imbalance.

We have revised the paper to emphasize this point more clearly at line 77. We appreciate the reviewer’s comment on the challenge of handling imbalanced data in machine learning. However, the main contribution of our study is exactly to propose a novel machine learning approach based on Multi-Objective Symbolic Regression (MOSR) that can effectively deal with imbalanced problems without altering the real distribution of the data in the population. We believe that this is a significant advantage over traditional machine learning models, especially for phenomena such as BSI/AMR that are naturally imbalanced and require accurate prediction of rare events. Our study demonstrates the superior performance of MOSR in minimizing false negatives and its potential for large-scale implementation to guide antimicrobial stewardship.

- The study lacks external validation of the MOSR models, which is necessary for assessing their real-world applicability.

We have clarified this point in the paper and added a discussion on the need for external validation in future work. We acknowledge the reviewer’s concern about the lack of external validation of the MOSR models, which is indeed a limitation of our study. However, we would like to emphasize that our study is a preliminary, exploratory, and innovative work that introduces a new machine learning approach for solving complex prediction tasks such as BSI/AMR. Our study is not intended to provide a definitive solution, but rather to demonstrate the feasibility and potential of MOSR for this domain.

- While the study emphasizes the interpretability of MOSR, the complexity of the models used, and their interpretability in a clinical setting are not thoroughly addressed.

We agree that this is an important aspect of the use of mathematical modelling in clinical analysis. The way we envision to achieve such interpretation in MOSR would be by analysing individual components of the expressions and, together with clinical expertise, contextualize the distribution and the mathematical relationships into the structure of the population of patients; finally, finding recurring patterns between the MOSR models we could assert specific associations that may be non-linear and complex. This is still an open research question and methodological challenge, we decided to leave this topic out of the scope of the paper.

- The study's reliance on a relatively small and specific dataset might not adequately represent the diverse scenarios encountered in different healthcare settings.

This is an important point. We have now addressed it in the paper at line 91 by providing more information on the dataset used. We have specified that the dataset was generated by a major University hospital in the UK and its ICUs, it spans 8 years of ICU admissions and with a heterogeneous population of patients. We have also discussed the challenges and opportunities for applying MOSR to other datasets and settings.

- Concerns about the class imbalance and lack of external validation might limit confidence in the study’s findings.

We have revised the paper at line 318 to clarify this point and to acknowledge the need for external validation in future work. We appreciate the reviewer’s attention on the class imbalance and the lack of external validation. However, as we have explained in the previous points, the main goal of our study is to introduce a novel machine learning approach based on MOSR that can effectively handle imbalanced problems without altering the real distribution of the data in the population. Therefore, we are not claiming that our study provides definitive clinical findings, but rather that it demonstrates a new methodology that can be applied to this class of problems.

- The study does not sufficiently address how its findings can be practically implemented in real-world clinical settings, especially considering the complexity of the models.

We have improved the paper at line 159 by providing more details on how the MOSR models can be integrated into clinical workflows and decision support systems. We have also emphasized that one of the unique features of MOSR is that the models are plain expressions that can be transferred to any information system (even a simple spreadsheet) and therefore their technical implementation is much simpler than any other alternative of ML. We have provided examples of how this can be done. We do not expect any practitioner to actually read the models in their practice, as they shouldn’t read a deep neural network, but only its output result. We envision the reading of the mathematical expressions limited to knowledge generation for the understanding of complex interaction between variables in a research environment, not in deployment.

- The work should expand the dataset to enhance representativeness, address data limitations, refine methodology, and improve model robustness and interpretability for practical clinical use.

We have improved the paper at line 90 by providing more details on the dataset used in our study. We have explained that the dataset represents the general population admitted to the ICU of a major UK hospital, which has been reduced due to data quality and completeness reasons. We have also discussed the limitations and challenges of the dataset, and how we have addressed them in our methodology. We have stated that this is standard practice, but we aim at expanding the representativeness of the dataset in future studies. We have also clarified that our study is a methodological proof more than the generation of clinical knowledge. We have emphasized the novelty and significance of our study in introducing a new machine learning approach based on MOSR that can effectively handle imbalanced problems without altering the real distribution of the data in the population.

###################################################

Reviewer #2: I don't have access to specific questions or answers related to a review. If you have any specific questions or content related to a review that you'd like assistance with, please provide more details, and I'll do my best to help.

###################################################

Reviewer #3: The submitted manuscript aims to analyze and predict bloodstream infections (BSI) and antimicrobial resistance (AMR) using machine learning (ML) techniques including Logistic Regression, Decision Trees, Random Forests, and symbolic regression.
I have few points to raise:

- The definition of AMR, authors stated that the definition of AMR line 111, “AMR was the culture presenting at least one resistance to ABs.” I don’t agree with definition, it should be more like “AMR was the culture presenting at least one resistance to Abs that once was successful treating the microbe”.

In line 111 we revised the definition of AMR to incorporate the comment to achieve a better definition.

- Please provide more data regarding AMR if available, the frequency of AMR is quiet high , 11 compared to 13 BSI in the test data reflecting high resistance that could be the definition used.

We defined AMR as the presence of any resistant microorganism in the blood cultures. Due to the scarcity of data representation, we couldn’t dive into them individually; this work is meant to show a first attempt to address the problem of AMR prediction in a sophisticated way leaving the door open to expand towards a more tailored analysis of individual microorganisms in future works.

- Please provide the 25 features used in the analysis in the appendix.

We provided the list of features with their distribution over the population.

- Is there any reason the authors choose this split training 90%, test 10% which deviates from normal ML approaches.

This was our reporting mistake, confused in the write up with the 10-fold cross validation of the training. We corrected the statement specifying that the train/test split was 80/20%.

- Looking at the symbolic regression equations, it seems the variables are interesting and the authors may provide explanation on them, example bilirubin reflecting liver injury, urea reflecting kidney injury along with temperature and mean arterial pressure, but my main concern is the number of antibiotics and the use of nystatin which primarily reflect higher clinical suspicious of BSI and AMR rather then actual predictive variable as it reflects the physician response to the presentation rather then the presentation itself. Alternative more suitable variable would be recent antibiotic use before the presentation.

This is a valid point, however, we are working with hospital data at presentation which does not necessarily have recent antibiotic use. From a methodological point of view this is a trade-off, and restricting ourselves to a smaller number of variables ensures a wider applicability of the model, such as for patients with limited clinical records – common scenario in ICU. The 25 we used are the result of running feature selection on 100+ variables in the training set prior to the full ML experiment. The clinical interpretation of our models is in the scope of future dedicated studies as it requires more methodological enquire.
