# Supplementary material for: Using interpretable machine learning to predict bloodstream infection and antimicrobial resistance in patients admitted to ICU: Early alert predictors based on EHR data to guide antimicrobial stewardship
Source: PLOS Digit Health. 2024 Oct 16;3(10):e0000641. doi: 10.1371/journal.pdig.0000641 (PMC11482717; doi:10.1371/journal.pdig.0000641)
Supplement: Attachment — Submitted filename: Response to Reviewers 2.docx [file pdig.0000641.s005.docx]

Dear Dr Chaurasia,

Thank you for providing us with valuable feedback on our paper: PDIG-D-23-00418. We appreciate the opportunity to address the concerns raised and to clarify aspects of our research.

# Reviewer 1

**Suggestions for the abstract**

1. ***Consider incorporating a statement on the innovative nature of the MOSR approach to highlight its importance more effectively.***

*We addressed this by adding line 38:*

*“an evolutionary approach to create ML models in the form of readable mathematical equations in a multi-objective way to overcome the limitation of standard single-objective approaches”*

2. ***Please provide a specific metric or statistic that quantifies the performance improvement of MOSR compared with traditional machine learning models.***

*We addressed this by adding lines 45-46*

*“with F1-Score .22 and .28 higher than any other alternative”*

3. ***Consider adding a sentence or two to the potential limitations or future directions for research to provide a more comprehensive overview.***

*We addressed this by improving lines 48-50*

*“Notably, the MOSR approach can be readily implemented on a large scale, offering a new ML tool to find solutions to these critical healthcare issues affected by limited data availability.”*

4. ***Use a more active voice throughout the abstract to make it more engaging and dynamic.***

Thank to your advice, the reviewed abstract sounds more engaging and appealing to the readers by pointing out a more detailed description of the MOSR methodology.

5. **Could you clarify the advantages of the MOSR approach over conventional machine learning techniques in diagnosing bloodstream infections and Antimicrobial Resistance? Furthermore, what might be the ramifications of this method in clinical practice and patient outcomes?**

In lines 44-47 we addressed this issue by being more specific.

“*In contrast, MOSR, specifically configured to minimize false negatives by optimizing also for the F1-Score, outperforms other ML algorithms”*

**Suggestions for the Introduction**

1. ***Consider adding 1-2 sentences summarizing the study objectives and anticipated contributions/findings.***

This is now stated in line 103

“Particularly, the choice of MOSR lies on its multi-objective capabilities and the ability to induce the model’s training to optimize also the F1-Score together with standard Binary Crossentropy. By choosing MOSR as ML algorithm we expect to replicate the same behaviour seen in literature and obtain a set of models able to produce more balanced performance metrics and put more attention in the least represented of the two classes.”

2. ***Specify whether "AMR" refers only to antibiotic resistance or includes other multidrug resistance as well.***

This is now stated in line 161.

“AMR is a positive blood culture presenting resistance to at least one antimicrobial medicines”

3. ***How can we tackle the pressing issue of Antimicrobial Resistance in Intensive Care Units, where the incidence of bloodstream infections reaches a staggering 35%?"***

*We are aware of the severity of this phenomenon, and as briefly described in lines 84-86, a multidisciplinary work is needed to tackle this problem. The contribution of this work is to provide a new improved tool for clinicians to be aware of which subset of patients are at higher risk of being infected with AMR which one day could be integrated with the current guidelines.*

**Suggestions for the Methodology**

1. ***Could you elaborate on the methods employed to confirm the discriminatory nature of the features identified via the HSIC Lasso, as it is imperative to ascertain their predictive capabilities and exclude the possibility of overfitting? Furthermore, incorporating an internal validation technique while selecting features would enhance the credibility of claims regarding their relevance.***

*We added more details in line 148*

*“*Only the features having a HSIC score larger than 1e-3 are used in the ML algorithms.”

2. ***Did you explore techniques to address the class imbalance problem beyond simply weighting the loss function? The results can be improved by addressing the imbalance more aggressively during model training.***

We address this in lines 222-224

We understand the importance of address the problem of class imbalance and this is why we implemented a solution using SMOTE and included the performance of that version of the ML models in the results table. This shows how re-balancing the classes worsen the performance of the model. Moreover, MOSR with the optimization of F1-Score was selected exactly to tackle a ML task with intrinsic class imbalance and, indeed, proved to produce better performance than other ML algorithms.

3. ***What methods will be employed to validate and interpret MOSR formulas, and how will clinicians/experts in the field utilize the gathered feedback to determine the applicability and generalizability of the findings?***

The predictive models produced by MOSR are like any other algorithm and could be used to generate either probability of belonging to the two classes or the binary classification itself. The formulas produced by these two experiments are overly complicated to be shown in a paper and to be read by humans, much like any other ML algorithm beyond the logistic regression.

The clinical evaluation will require a dedicated work exploring the meaning of each individual feature used by the MOSR algorithm in this case, and it is listed from line 312 as one of the future developments required in this research endeavour.

4. **"The MOSR approach is characterized by innovation, yet it could be further enhanced through the incorporation of established machine learning methodological practices, such as validation, class imbalance mitigation, and clinical evaluation."**

We agree on this claim and we included its message in line 312.

**Suggestions for the Result section**

1. ***Provide more details on the epidemiological analysis, such as the time period and number of patients. Clarify if Figure 2 is from the study data or other sources.***

This has now been clarified in line 243 an 245.

“This retrospective study uses data of 1142 patients collected from the ICUs within Guy's and St. Thomas' NHS Foundation Trusts in Central London during this specific period, we conducted a detailed examination of this intriguing phenomenon. All patients of at least 18 year of age at their admission to ICU are included in this dataset.”

2. ***For Table 1, report statistical tests used to compare groups (e.g., t-tests, chi-sq) and indicate which features showed significant differences.***

This has been added in line 264

“The statistical relevance is assessed by means of the appropriate statistical test based on the data type and distribution (t-test for the continuous variables, ANOVA for categorical variables).”

3. ***Undertake further evaluation using additional metrics beyond AUROC/accuracy, such as precision-recall curves and F1 score plots at various thresholds, particularly for imbalanced classes.***

Detailed performance metrics are reported in Tables 2 and 3, and because the algorithms are trained with the standard threshold at 0.5, any other threshold would not improve the overall metric performance. The quality of the models in this imbalanced datasets is assessed by means of F1-Score that reaches its highest value with MOSR.

4. ***Please present the top-performing formulas for the MOSR results to highlight their interpretability. In addition, we evaluated the feature importance derived from these formulas.***

The formulas generated by MOSR in this instance are reported in Appendix A for a selection of models that are simple enough to be readable. The importance of each feature fall into the clinical evaluation of the model that is purposely left out of the scope of this work as this is a proof-of-methodology to tackle this class of ML tasks in a novel, more effective way.

5. ***Discuss the limitations of the MOSR approach for this problem, for example, potential for overfitting, handling class imbalance, and generalizability with limited data.***

*We addressed this point in line 304*

*“*The improved performance of MOSR on the test sets are an indication of its ability to mitigate overfitting behavior.”

MOSR is a methodology that has been chosen to better handle class imbalance and its superior test performance show how its ability to mitigate overfitting and improve generalizability.

6. ***Expand discussion of related work, comparing data/models used. The limitations of this study should be addressed in future research.***

The discussion of related work take into account all the published studies that produce comparable methodologies and are described in detail from line 353.

More details on the comparison with literature has been provided in line 369.

“Overall, the reviewed approaches make use of variables that are not readily available at patient admission in their EHR records, which was a core study design choice of our proposal. Moreover, this work is the first of its kind proposing the introduction of the methodology of MOSR and multi-objective training.”

7. ***Suggest validation of new prospective data to assess generalizability and clinical utility before deployment.***

As described in line 393, generalizability is one of the main strength points of MOSR although it requires further external validation in order to be of sufficient quality and clinical utility for deployment.

**General Question**

All these questions were already addressed previously.

1. What was the main goal of this study described in the paper?
2. What is Symbolic Regression, and how does it differ from other machine learning algorithms?
3. What is the advantage of using Symbolic Regression in this study?
4. How does this study address the class imbalance issue in a dataset?
5. What is the significance of prioritizing sensitivity in this study?
6. How do the authors validate the performance of machine-learning models?
7. What are the limitations of this study owing to the dataset size?
8. How can we enhance these models in future research?
9. Could you elaborate on the possible clinical implications of the predictive models developed in this study?
10. What is the significance of this research in predicting bloodstream infections and antimicrobial resistance, and how does it relate to existing studies on the topic?

# Reviewer 2

**Recommendations for Improvement:**

1. ***Literature Contextualization: Enhance the introduction by including a brief review of similar studies to establish a more defined research gap.***

Thank you. We conducted a literature review of similar applications and described it the final discussion (line 324) to create an improved narrative flow.

2. ***Dataset Details: Provide more specifics about the initial dataset selection and the types of clinical data used.***

The dataset selection is outlined from line 243. The type of clinical data used is described with detailed distribution in Table 1.

3. ***Model Performance Analysis: In the results section, include a deeper analysis of why MOSR performed better, focusing on the features and model characteristics that contributed to its success.***

The equations of the models produced are all presented in Appendix A. Their full clinical interpretation requires deeper methodological theory of non-linear equations and their relationship with real-world phenomena, which is out of scope of this paper. We detailed the better performance of MOSR in line 309.

4. ***Comparative Discussion: Compare the findings with other similar studies in the discussion to contextualize the study's contribution to the field.***

The discussion of related work takes into account all the published studies that produce comparable methodologies and are described in detail from line 353.

5. ***Implementation Insights: Discuss the practical challenges and potential strategies for implementing the proposed ML model in real-world clinical setting***

The limitations of this work are outlined in the final part of the discussion (line 381), where we highlight the need for a larger and more representative dataset and the identification of more specific target outcomes relating to individual microorganisms. Regarding the implementation in real-world clinical setting, further consideration has been added in line 374.

6. ***Expand Literature Review: Include a more extensive review of previous studies using ML for BSI and AMR prediction. This could provide a broader context and justify the study's novel contribution more effectively.***

*The literature review was conducted on Pubmed, Google Scholar and Scopus, aiming at finding all the papers that made use of innovative ML methodologies to predict ARM in ICU. The inclusion of the papers in our comparison was based on the similarity to our approach. This is now detailed in line 353.*

7. ***Detailed Methodological Justification: While the methodology is robust, a more detailed justification for the choice of specific ML models and their configurations would add depth to the understanding of their selection.***

In line 174 we added clarification on the configuration of the algorithms.

“For all algorithm, a comprehensive grid search optimization of hyperparameters has been conducted during training.”

8. ***Enhanced Discussion on Model Interpretability: Given the emphasis on interpretability, a deeper discussion on how MOSR facilitates clinical decision-making compared to less interpretable models would be beneficial.***

The formulas generated by MOSR in this instance are reported in Appendix A for a selection of models that are simple enough to be readable. The importance of each feature fall into the clinical evaluation of the model that is purposely left out of the scope of this work as this is a proof-of-methodology to tackle this class of ML tasks in a novel, more effective way.

9. ***Addressing Data Limitations: • Future research directions could include strategies to overcome the data limitations noted, such as collaborating with other institutions for a more extensive dataset.***

We have now referred to the use of Federated Learning strategies as a mitigation approach for such a problem. Line 384

10. ***Practical Implementation: • Discuss the practical aspects of implementing the MOSR model in clinical settings, including integration with existing healthcare IT systems and clinician training***

We added deeper consideration in line 390

“The third limitation relates to the need to identify the most effective and reliable representation of the predictions for a proper integration with clinical decision support systems. This would require a closer work with practicing clinician to understand the nuances of their decision-making environment and what type of representation would convey the prediction best. “

11. ***Incorporate Latest Research References: Update the text with the latest references and studies in the field of machine learning applications in healthcare. This will ensure that the content remains current and relevant.***

*The latest literature review is dated April 2024*

12. ***Detailed Explanation of MOSR: Expand on the explanation of Multi-Objective Symbolic Regression (MOSR), possibly including a brief overview of its mechanics and advantages over traditional models. This would help readers understand why MOSR is more effective.***

*The detailed mechanisms of MOSR are outlined in line 178*

13. ***Patient Impact Focus: Include a section or paragraph that specifically discusses the impact of this research on patient outcomes. This can help to humanize the data and emphasize the real-world implications of the findings.***

This aspect is discussed in the Discussion and Conclusion section in line 325
